# *GhTGA2*, a Potential Key Regulator of Salt Stress Response: Insights from Genome-Wide Identification of *TGA* Family Genes Across Ten Cotton Species

**DOI:** 10.3390/genes16101143

**Published:** 2025-09-26

**Authors:** Lu Meng, Jiliang Fan, Shandang Shi, Faren Zhu, Ganggang Zhang, Junwei Wang, Zihan Li, Fei Wang, Hongbin Li

**Affiliations:** 1 Key Laboratory of Oasis Town and Mountain-Basin System Ecology of Xinjiang Production and Construction Corps, Key Laboratory of Xinjiang Phytomedicine Resource and Utilization of Ministry of Education, College of Life Sciences, Shihezi University, Shihezi 832003, China; mlulu@stu.shzu.edu.cn (L.M.); fanplanbeson@163.com (J.F.); shi_shandang@163.com (S.S.); zhufaren163@163.com (F.Z.); 19915233386@163.com (G.Z.); 18287018531@163.com (J.W.); feiw@shzu.edu.cn (F.W.); 2 Department of Civil, Environmental and Construction Engineering, College of Engineering and Computer Science, University of Central Florida, Orlando, FL 32816, USA; zihl2721@gmail.com

**Keywords:** cotton, TGA gene family, VIGS, salt stress

## Abstract

Background: The *TGACG-BINDING FACTORS (TGA)* gene family, a key subgroup of bZIP transcription factors, mediates plant stress responses and developmental processes by binding to the *as-1 cis*-element in target gene promoters to regulate transcriptional activation or repression. Despite its functional significance, systematic characterization of *TGA* genes in cotton (*Gossypium* spp.) remains insufficient. Methods: In this study, we performed a comprehensive genome-wide identification and phylogenetic analysis of *TGA* members across 10 *Gossypium* species and verified the functions of candidate genes using VIGS technology. Results: A total of 74 *TGA* homologous genes with conserved DOG1 and bZIP domains were identified. Evolutionary analysis revealed that the cotton *TGA* gene family can be classified into five distinct branches, suggesting functional diversification. Functional prediction analyses indicated these genes in cotton growth regulation and stress adaptation, potentially through hormone-mediated signaling pathways. Expression profiling demonstrated both tissue-specific expression patterns and salt-stress responsiveness in *Gossypium hirsutum TGA* genes, and *GhTGA2* exhibited the most significant up-regulated expression under salt stress. Virus-induced gene silencing (VIGS)-mediated *GhTGA2* silencing significantly reduced the salt tolerance in cotton. Conclusions: The *TGA* gene family is involved in regulating cotton growth, development, and stress responses, and plays a critical role in mediating salt stress tolerance in cotton. Our results provide mechanistic insights into cotton stress adaptation and establish a valuable genetic resource for developing elite salt-tolerant cotton cultivars, with direct implications for sustainable cotton production.

## 1. Introduction

Cotton is a significant economic crop cultivated extensively across the globe. As arable land continues to diminish, optimizing the use of saline-alkali land for cotton cultivation becomes crucial for ensuring the sustainable development of cotton production [1]. Cotton exhibits moderate salt tolerance, with sensitivity varying across growth stages; notably, the seed germination and seedling phases are particularly sensitive to salt stress [2]. The salt tolerance in cotton is a complex quantitative trait controlled by multiple genes and can be significantly influenced by environmental factors [3]. *G. hirsutum* represents the most widely grown variety worldwide, accounting for approximately 95% of global cotton planting area. However, current commercial *G. hirsutum* cultivars exhibit limited salt tolerance, rendering them inadequate for cultivation in saline-alkali environments [4]. Furthermore, soil salinization poses a global ecological challenge that hampers sustainable agricultural development and has resulted in substantial yield reductions on nearly one-third of irrigated lands worldwide [5]. Salt stress induces multifaceted damage in plants, primarily including osmotic stress, ion toxicity, and subsequent oxidative stress among others [6]. Consequently, identifying salt-tolerant genes within *G. hirsutum* and elucidating its salt-adaptation mechanisms are essential steps toward breeding resilient cotton varieties capable of enhancing yields in saline-alkali conditions.

The bZIP (basic region/leucine zipper) transcription factor family is a prevalent class of transcription factors in plants, characterized by its conserved bZIP domain [7]. This bZIP domain consists of 60–80 amino acid residues and comprises a highly conserved basic amino acid region (basic region, BR) as well as a leucine zipper region (Leucine zipper region, LZ) [8]. By interacting with the core sequence ACGT located in the promoter regions of target genes, these factors regulate the expression of downstream genes, thereby executing their biological functions [9]. Based on structural characteristics and variations in the *cis*-acting elements they recognize, the bZIP transcription factor family can be categorized into ten subfamilies (A–I and S) [10]. These subfamilies exhibit evolutionary conservation throughout evolution while each possesses distinct functional attributes. These proteins regulate critical biological processes during various stages of plant growth and development, and also contribute to adaptive responses to biotic pathogens and abiotic environmental challenges [11]. Notably, members of the D subfamily are capable of binding to the activating sequence 1 element (*as-1*, 5′-TGACGTC-3′) within the promoter region of the *Nicotiana tabacum* pathogenesis-related protein gene *PR-1a*; consequently, they are also designation TGACG-BINDING FACTORS (TGA) [12,13].

TGACG-binding (TGA) transcription factors are among the earliest studied plant transcription factors, first identified and characterized in *N. tabacum* in 1989 [14]. Currently, members of the *TGA* gene family have been identified and extensively studied across various model plants, including *A. thaliana*, *N. tabacum*, *Zea mays*, *Cucumis melo*, *Helianthus annuus*, *Citrus sinensis*, *Brassica napus*, *Arachis hypogaea*, *Carica papaya*, and *Musa acuminata* [15,16,17,18,19,20,21,22,23,24]. In dicotyledonous plants specifically, ten *A. thaliana TGA* gene family members—*AtTGA1–7*, *AtPAN* and *AtTGA9–10*—have been investigated most comprehensively [25]. In *A. thaliana* based on amino acid sequence similarity; TGA transcription factor family members can be categorized into five distinct groups: group I comprises *AtTGA1* and *AtTGA4*; group II includes *AtTGA2* as well as *AtTGA5* and *AtTGA6*; group III consists of *AtTGA3* and *AtTGA7*; group IV encompasses both *AtTGA9* and *AtTGA10*; while group V is represented solely by *AtPAN* (PERIANTHIA) [26]. Notably, TGA transcription factors from different groups exhibit specific structural and functional differences. Groups I through III contain typical bZIP domains that enable interaction with NONEXPRESSOR OF PATHOGENESIS-RELATED GENES 1 (*NPR1*), thereby participating in the plant’s disease resistance response [27]; conversely groups IV and V possess relatively unique structures that play crucial roles in regulating flower organ development among other functions [28]. The *TGA* gene family exhibits a similar classification method across various plant species. In *Z. mays*, the *ZmTGA* gene family can be categorized into five distinct groups [17]. The *MaTGA* gene family in *M. acuminata* is divided into four subgroups [18]. Additionally, seven *CsTGAs* genes have been identified within the *C. sinensis* genome and classified into five subgroups [21]. In *Oryza sativa*, there are ten members of the TGA transcription factor family, which can also be organized into five groups and exhibit a high degree of homology with the *A. thaliana* TGA transcription factor family [29]. The *TGA* gene family represents a crucial component of the bZIP transcription factor family, playing significant roles in plant growth and development as well as responses to both biotic and abiotic stresses. This is achieved through their involvement in hormone response pathways that regulate plant resistance or flower organ development [30]. Research has demonstrated that overexpression of the *GmTGA26* gene in *G. max* can alleviate membrane oxidative damage to cell membranes caused by salt stress in soybean leaves while significantly enhancing salt tolerance in *G. max* plants [31].

Currently, there is a paucity of studies focusing on the TGA transcription factor family in cotton. To date, only one *GhTGA2.2* gene has been cloned in research pertaining to cotton’s response to *Verticillium dahliae* [32]. Furthermore, the genome-wide characteristics of the TGA transcription factor family in *Gossypium* and its regulatory mechanisms under salt stress remain largely unexplored. In this study, we performed a comprehensive genome-wide analysis of the TGA transcription factor family across ten cotton varieties—comprising five diploid (A/D genomes) and five allotetraploid (AD genomes) species—for the first time. Utilizing whole-genome data, we successfully identified members of the *TGA* gene family within each *Gossypium* variety. Phylogenetic analysis elucidated evolutionary relationships between cotton TGA factors and their orthologs in *A. thaliana* (AtTGAs) and *Theobroma cacao* (TcTGAs). Additionally, detailed information regarding chromosomal locations, gene structures, conserved motifs, promoter *cis*-acting elements, and physicochemical properties of the *TGA* gene family in cotton were thoroughly examined. Subsequently, we investigated tissue-specific expression patterns and expression profiles of the *TGA* gene family in *G. hirsutum* subjected to various stress conditions through transcriptome data analysis. *GhTGA2* was ultimately selected as a candidate gene for further investigation. To validate its function under salt stress conditions in cotton, we employed VIGS technology to silence the *GhTGA2* gene in *G. hirsutum*. This study aims to elucidate the regulatory signaling pathways involved in the response of TGA transcription factors to salt stress in cotton, ultimately identifying key genomic regions that can be targeted for developing stress-tolerant varieties via molecular breeding strategies.

## 2. Materials and Methods

### 2.1. Identification of TGA Gene Families

The gene annotation files, gene sequences, and protein sequence data for both diploid and tetraploid cotton species were obtained from the Cottongen database [33]. Additionally, biological information for the cotton-related species *T. cacao* was obtained from NCBI. The genome annotation and sequence data of *A. thaliana* utilized in this study were sourced from the TAIR database [34]. Blastp alignment was conducted using TBtools software (v2.210) [35] to identify candidate members of the *TGA* family in cocoa and ten cotton varieties. Furthermore, conserved protein domains were analyzed using NCBI’s CD-Search function to ensure that identified protein sequences contained DOG1 and bZIP domains. The number of *TGA* family members was determined accordingly.

### 2.2. Chromosomal Localization and Properties of Proteins of the TGA Family

Based on the *TGA* family genome annotation file, identify the seat numbers of *TGA* genes and utilize TBtools (v2.210) to extract the chromosome lengths for *A. thaliana*, *T. cacao*, and 10 *Gossypium* species. Next, utilize the online tool MG2C_v2.1 to map the exact chromosomal positions of the *TGA* gene family members [36].

### 2.3. Phylogenetic and Collinearity Analysis of TGA Gene Families

In this study, we conducted phylogenetic analyses of the protein sequences encoded by the *TGA* gene family in *Gossypium*, *A. thaliana*, and *T. cacao.* The data analysis was conducted through multidimensional alignment of protein sequences employing the ClustalW alignment tool integrated within the MEGA7.0 (v7.0.26) software suite. Phylogenetic relationships were reconstructed utilizing the Neighbor-Joining algorithm, with 1000 bootstrap iterations implemented for node support evaluation [37]. The generated phylogenetic tree was visualized using TBtools (v2.210) software and plotted utilizing the online mapping site iTOL [38]. Furthermore, we prepared a genomic annotation file for the cotton *TGA* gene family and utilized TBtools (v2.210) software to analyze collinearity relationships among members of this gene family throughout evolution [39].

### 2.4. Genomic Architecture, Evolutionarily Conserved Motifs, and Structurally Preserved Domains in Cotton TGA Family

The exon–intron architecture was graphically represented using the Gene Structure Visualization module within TBtools (v2.210), based on annotated genomic sequences derived from the *Gossypium* TGA transcription factor group [35]. The MEME Suite [40], Pfam, PROSITE, and Motif Scan were utilized for predicting conserved protein sequences and analyzing domain structures. Additionally, ProtParam online tools were employed to assess the physical and chemical properties of cotton *TGAs* [17].

### 2.5. Identification of cis-Regulatory Motifs in Gossypium TGA Transcription Factor Promoters

The 2000 bp region upstream of the cotton *TGAs* sequence was selected as the promoter region for the *Gossypium TGAs* gene. This sequence was uploaded from PlantCare for analysis of promoter *cis*-elements. The results of this analysis were visualized using TBtools (v2.210) [41].

### 2.6. Expression Patterns of the TGA Gene Family in G. hirsutum and Their Responses to Stress

In order to investigate the tissue-specific expression of *TGA* family genes in *G. hirsutum* and their distinct response patterns to various stressors, we analyzed and processed published transcriptome data under different conditions. Transcriptome data for *G. hirsutum* tissues and abiotic stress were downloaded from the NCBI SRA website, and the genome sequencing project with accession number PRJNA248163 [42]. This investigation allowed us to determine the expression patterns of TGA transcription factors in distinct tissues and organs of *G. hirsutum*, while also elucidating their regulatory dynamics under various abiotic stress conditions, including low temperature, thermal stress, water deprivation, and salinity exposure. Heat maps illustrating these expression profiles were generated using TBtools (v2.210).

Download the coding sequence (CDS) information for the *TGA* gene of *G. hirsutum* from the Cotton MD database. Subsequently, design specific fluorescence quantitative PCR primers related to this gene using Prime 5.0 software (Table S2). Revised version: Total RNA isolation was performed with the RNAprep Pure Polyphenol-Polysaccharide RNA Extraction Kit, followed by complementary DNA synthesis using reverse transcriptase. *GhUBQ6* served as the constitutive control for normalization. Four-week-old *G. hirsutum* plants underwent sodium chloride (NaCl) treatment (200 mM) with subsequent sampling at 60, 180, 360, and 720 min intervals post-exposure [43]. Subsequent to experimental interventions, RNA specimens underwent reverse transcription to generate cDNA templates for real-time quantitative PCR (qPCR) profiling. The amplification protocol comprised (1) preliminary denaturation at 95 °C (5 min); (2) 40 repetitive cycles incorporating 95 °C denaturing phase (30 s), and 60 °C primer hybridization/elongation stage (60 s). Transcript quantification was executed via the 2^−ΔΔCt^ algorithm with triplicate experimental replicates ensuring analytical reproducibility.

### 2.7. Subcellular Localization of the Candidate Gene GhTGA2

Primer design was conducted using Primer 5.0 software (Table S2), incorporating *Kpn* Ⅰ (GGTACC) and *Xba* Ⅰ (TCTAGA) restriction sites at the 5′ ends of the upstream and downstream primers, respectively. The target fragment, approximately 1390 bp in length, was PCR amplified utilizing cDNA from *GhTGA2* as a template. Following sequencing verification of the amplified gene, the *GhTGA2* gene fragment was inserted into the expression vector *pCAMBIA1300-eGFP*. The recombinant vector *pCAMBIA1300-eGFP:GhTGA2* was subsequently obtained after digestion verification. The constructed *pCAMBIA1300-eGFP:GhTGA2* vector was transformed into *A. rhizogenes* GV3101 and infiltrated into the leaves of four-week-old *N. tabacum* plants (*N. benthamiana*). The plants were then incubated in darkness for 48 h to facilitate transformation. Epidermal fluorescence emission patterns in *N. benthamiana* foliar specimens were acquired through laser-scanning confocal imaging (Nikon A1R HD25, Tokyo, Japan) under standardized excitation/emission parameters [44]. Agrobacterium tumefaciens cultures carrying engineered plasmid constructs were propagated in Luria–Bertani (LB) broth under selective pressure. The *pCAMBIA1300-35S-mCherry-NLS* vector (Puint Biotech, Taiyuan, China) was employed as a nuclear-targeted fluorescence reporter system throughout the experimental workflow.

### 2.8. Functional Validation of GhTGA2

To functionally characterize the saline stress resilience conferred by *GhTGA2*, a *N. tabacum* rattle virus (TRV)-mediated gene silencing platform was implemented to achieve targeted transcriptional knockdown of *GhTGA2* in *G. hirsutum* cultivars. Primer 5.0 software was used for primer design (Table S2), and *EcoR* Ⅰ (GAATTC) and *Xma* Ⅰ (CCCGGG) restriction sites were added to the 5′ ends of the upstream and downstream primers, respectively. The VIGS-specific fragment (about 325 bp) was amplified. After sequencing verification of the amplified gene, the gene fragment was inserted into the silencing vector *TRV2*, and the recombinant vector *TRV2:GhTGA2* was obtained after enzyme digestion verification. Meanwhile, the *TRV2:GhPDS* vector was constructed as a positive control. The correctly constructed silencing vectors were introduced into Agrobacterium LBA4404 by electroporation. The transformed Agrobacterium was cultured to the logarithmic growth phase, and the appropriate infection concentration was adjusted (OD 600 = 1.2). *TRV1,* along with *TRV2* empty vectors and positive controls and *TRV2:GhTGA2*, were mixed in a 1:1 ratio and subsequently used to infect cotton leaves via injection. The infected cotton was dark-cultured overnight and then placed in a 25 °C cotton growth chamber with a light–dark cycle of 16 h:8 h.

Most studies have indicated that the optimal screening concentration for assessing salt tolerance in cotton is between 100 and 300 mM [43]. To mitigate the impact of soil matrix on salt concentration, we irrigated both the experimental plants and control plants with a 200 mM NaCl solution as a whole. In contrast, the control group received an equal volume of distilled water. Subsequently, they were cultured under standard conditions, and phenotypic changes were monitored after a treatment period of 15 days.

### 2.9. Statistical Analysis

Biological datasets were subjected to parametric analysis using SPSS 29 (IBM, Armonk, NY, USA). Inter-group variance across temporal intervals and experimental conditions was determined through Duncan’s multi-comparison procedure. Statistical thresholds were operationalized as *p* < 0.05 (significant), *p* < 0.01 (highly significant), and *p* > 0.05 (non-significant), maintaining family-wise error rate control.

## 3. Results

### 3.1. Identification and Chromosomal Localization of the TGA Gene Family

**Figure 1:**
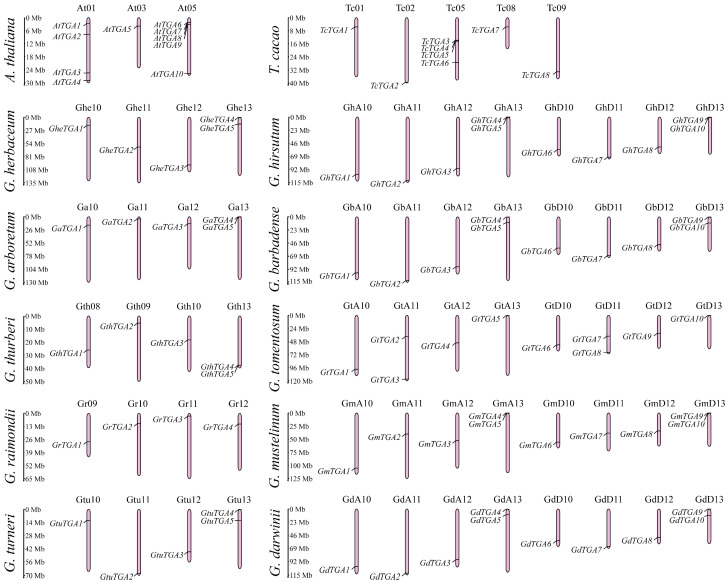
Chromosome localization of *TGA* gene family. Chromosomes are shaded in pink, individual *TGA* genes are marked, and chromosome names are labeled in dark. The vertical scale positioned along the left margin denotes chromosomal lengths in Mb.

In this study, the AtTGA protein sequence was utilized as a reference to screen for *TGA* homologous sequences within the cotton database through homology comparison. The results of protein conserved domain predictions were also integrated into this analysis. Following the screening of genomic sequences and the removal of redundant entries, a total of 74 members of the *TGA* gene family were identified in the cotton genome. Among these, 5 *TGA* genes were detected in each diploid *Gossypium* species, including *Gossypium herbaceum*, *Gossypium arboreum*, *Gossypium thurberi*, *Gossypium raimondii*, and *Gossypium turneri*. In contrast, each tetraploid *Gossypium* species—namely *G. hirsutum*, *Gossypium barbadense*, *Gossypium tomentosum*, *Gossypium mustelinum*, and *Gossypium darwinii*—harbored ten *TGA* genes. The naming convention for the *TGA* family genes was based on their chromosomal locations; they were designated as *AtTGA1–10* for *A. thaliana* (At), *TcTGA1–8* for *T. cacao* (Tc), *GheTGA1–5* for *G. herbaceum* (Ghe), *GaTGA1–5* for *G. arboreum* (Ga), *GthTGA1–5* for *G. thurberi* (Gth), *GrTGA1–4* for *G. raimondii* (Gr), and so forth, with corresponding designations such as *GhTGA1–10* and *GbTGA1–10* representing other species within this classification system. Based on the results of chromosome mapping, it can be concluded that *A. thaliana TGA* gene family is distributed across chromosomes 01, 03, and 05, with the most significant concentration found on chromosome 05. In contrast, the *T. cacao TGA* gene family is primarily located on chromosomes 01, 02, 05, 08, and 09, also exhibiting the highest number of genes on chromosome 05. The *TGA* family genes in *Gossypium* are predominantly clustered on chromosomes 10, 11, 12, and 13 across various *Gossypium* species. *G. thurberi* possesses one gene each on chromosomes 08 and 09 but lacks a gene on chromosome 11; similarly, *G. raimondii* has a gene situated on chromosome 09 but none present on chromosome 13. Notably, except for *G. tomentosum*, which contains two *TGA* family members located on chromosomes A11 and D11, the remaining seven cotton varieties exhibit two genes positioned on chromosome 13 while having only one gene each located on chromosomes 10, 11, and 12 (Figure 1).

The physicochemical properties of the *TGA* gene family, which includes *A. thaliana*, *T. cacao*, and *Gossypium*, were analyzed using TBtools (v2.210) software (Table S1). Biochemical profiling demonstrated a molecular mass spectrum spanning 26,271.35 to 59,078.52 Da across TGA transcription factors. Concomitantly, gene products derived from *TGA* homologs exhibited polypeptide chain length polymorphisms ranging between 235 and 532 residues. The predicted theoretical isoelectric points (PI) ranged from 5.34 to 9.62, while the instability index exceeded 40 for all members except *AtTGA10*, which had an index of 37.3; this suggests that most TGA family proteins are unstable. Furthermore, the Grand Average of Hydropathicity (GRAVY) values were negative across all members of the TGA family proteins, indicating their hydrophilic nature.

### 3.2. Evolutionary Relationship Analysis of TGA Gene Family

**Figure 2:**
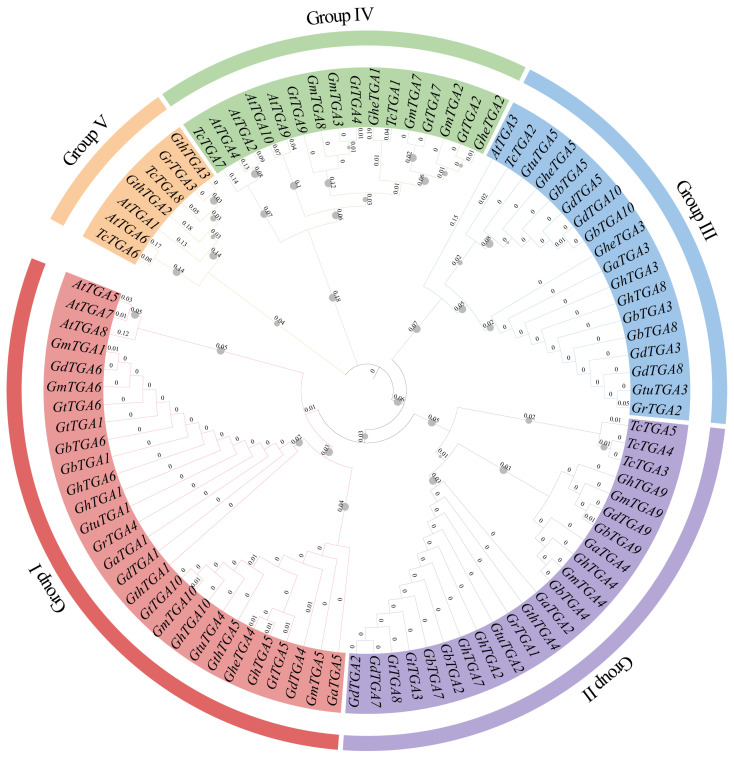
Phylogenetic and evolutionary relationship analysis of *TGA* gene family. Phylogenetic analysis of TGA proteins from 10 *Gossypium*, *A. thaliana*, and *T. cacao* species (different colored branches represent different sub-branches of the TGA protein family). The gray dots denote bootstrap.

To delineate evolutionary divergence patterns within the TGA transcription factor family, we conducted maximum likelihood phylogenetic reconstruction using protein orthologs from *A. thaliana* (*n* = 10), *T. cacao* (*n* = 8), and *Gossypium* species (*n* = 74), with branch topology detailed in Figure 2. Based on the phylogenetic analysis, the *TGA* gene family can be categorized into five subfamilies: Group I through Group V. Phylogenetic analysis of *Gossypium* and *T. cacao TGA* genes reveals a similar clustering pattern and high sequence homology with *A. thaliana*. The number of TGA proteins present in cotton remains remarkably stable: diploid cotton species possess five TGA proteins whereas tetraploid species contain approximately ten. This distribution corresponds well with established evolutionary patterns within cotton species. All genes demonstrate one-to-one homology between genomes or subgenomes—indicating that these genes may serve analogous functions across different varieties of *Gossypium*.

### 3.3. Chromosomal Collinearity Analysis of the TGA Gene Family in Gossypium

**Figure 3:**
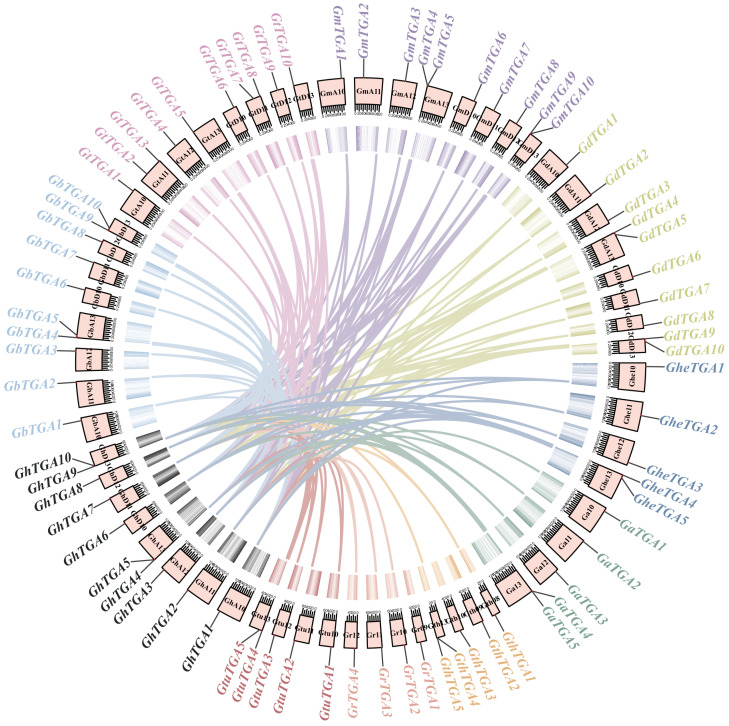
Collinearity between *G. hirsutum* and nine cotton species based on homologous gene pair analysis. Chromosomes are shown in pink, and collinear genes are connected by different colors. The genes and chromosome gene density are distinguished by different colors.

During evolution, chromosomal segment duplication across various genomes through fragment replication, tandem duplication, and whole-genome duplication (WGD) contributes to the expansion of gene families. While duplicated genes may initially exhibit functional redundancy, they frequently undergo subfunctionalization or neofunctionalization during divergence. To investigate the evolutionary relationships among gene families, we employed the TBtools (v2.210) with default parameters (blast E-value ≤ 1 × 10^−5^, window size = 10 genes) to perform a collinearity analysis between *G. hirsutum* and nine other *Gossypium* species. Syntenic analysis revealed significant lineage-specific divergence in orthologous *TGA* distributions: allotetraploid *Gossypium* species (e.g., *G. hirsutum–G. barbadense*) retained 24–30 inter-subgenomic (AD) ortholog pairs, compared to only 5–13 intra-genomic (A/D) pairs in diploids (e.g., *G. herbaceum–G. arboreum*), with >92% of syntenic *TGA* loci mapping to non-homologous chromosomes (Figure 3)—a genomic architecture signature strongly implicating segmental duplications, rather than whole-genome doubling, as the primary driver of *TGA* family expansion in *Gossypium*. The prevalence of interchromosomal synteny blocks, coupled with Ks divergence peaks corresponding to ancestral polyploidization events (~1.5 MYA for AD genomes; ~5 MYA for A/D diploid divergence), indicates that segmental duplications—potentially linked to transposon-mediated genome reshuffling—were pivotal for *TGA* diversification. Intriguingly, 68% of duplicated *TGA* genes showed conserved expression patterns (FPKM > 1), suggesting selective retention of functionally essential copies.

### 3.4. Analysis of the Gene Structure, Conserved Motifs, and Conserved Domains of the TGA Gene Family in Gossypium

**Figure 4:**
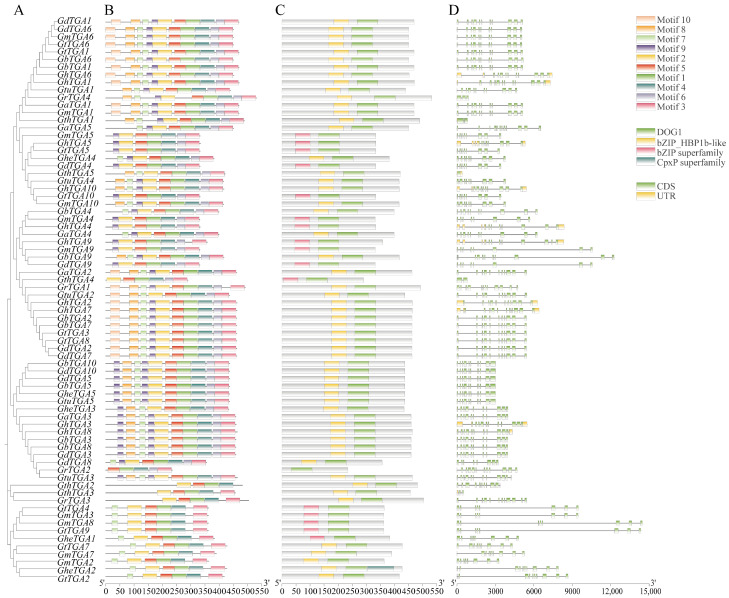
Phylogenetic tree, conserved motifs, conserved domains, and gene structure analysis of TGA proteins from 10 *Gossypium* species. (**A**) NJ tree constructed with TGA proteins. (**B**) Conserved motif analysis of TGA proteins. (**C**) Analysis of conserved domains of TGA proteins. (**D**) Analysis of gene structures of *TGA*.

Conserved gene sequences serve as fundamental markers in the study of molecular evolution. By examining the similarities and differences among these gene sequences and their corresponding structures, researchers can accurately infer phylogenetic relationships and evolutionary histories among species, identify homologous genes, and trace the expansion and functional differentiation of gene families [19]. In this study, we analyzed the gene structures, conserved motifs, and domains of the *TGA* gene families in 10 *Gossypium* species (Figure 4). The analysis revealed that most *TGA* genes consist of 8–12 exons; notably, 83.78% of these genes lack 5′/3′–untranslated regions (UTRs). Furthermore, genes within the same group exhibit similar exon–intron architectures. All ten *TGA* genes were found to contain non-coding introns. Amino acid sequence alignment analysis of conserved *TGA* gene domains in cotton indicated that all TGA proteins possess one bZIP_HBP1b-like domain along with one DOG1 domain. Additionally, some TGA proteins also include a bZIP superfamily domain and a CpxP superfamily domain. This suggests that they may have distinct functions compared to other family members. Application of the MEME algorithm (Multiple Expectation Maximization for Motif Elicitation) enabled systematic detection of 10 evolutionarily preserved sequence signatures. Phylogenetic analysis revealed that *TGA* homologs clustered within identical clades exhibited pronounced structural conservation patterns, suggesting biological role convergence. Notably, all 74 *Gossypium TGA* homologs maintained five core modular architectures (Motif 1–Motif 5) across taxonomic boundaries. Based on our constructed evolutionary tree for *TGA* family in *Gossypium*, it is evident that these proteins can be categorized into five subfamilies. Notably, Motif 10 is specific to subfamilies I and II in *Gossypium*; conversely, most members belonging to subfamily III harbor two instances of Motif 9—this distinction may significantly contribute to varying physiological roles exhibited by different subgroups within *Gossypium TGAs*.

### 3.5. Prediction of cis-Acting Elements in the Promoter Region of the TGA Gene Family of Cotton

**Figure 5:**
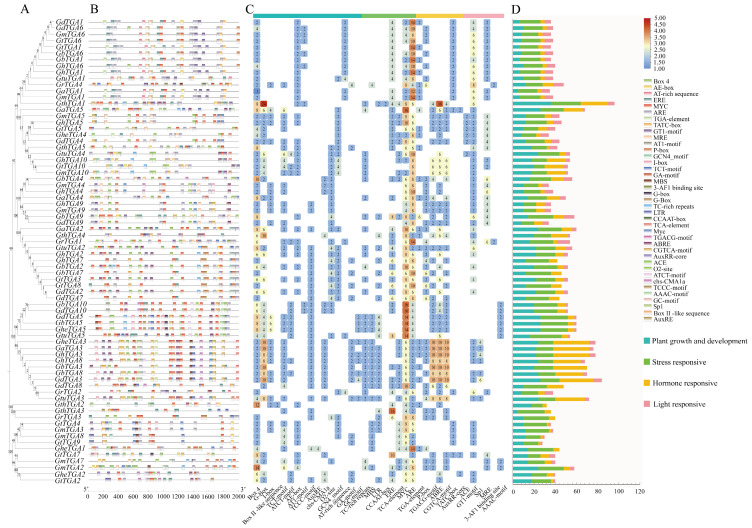
Prediction and analysis of *cis*-acting elements in the 2000 bp upstream promoter regions of the *TGA* gene family. (**A**) NJ tree constructed with TGA proteins. (**B**) The distribution of *cis*-acting regulatory elements in the 2000 bp upstream promoter regions of this gene family is shown in the figure using distinct colors and symbols, with a scale bar at the bottom indicating sequence orientation and length. (**C**) Statistical analysis showed the number of *cis*-acting elements in the *TGA* gene family, with deeper colors indicating a higher count of predicted elements. (**D**) Stacked histogram of the four types of *cis*-acting elements.

To delineate transcriptional regulatory mechanisms governing *Gossypium* TGA factors, we performed computational screening of promoter architectures using the PlantCARE database, revealing four phylogenetically conserved *cis*-regulatory element categories associated with hormonal response and abiotic stress adaptation as shown in Figure 5. Previous studies demonstrate that TGA transcription factors regulate diverse biological processes in plants, including developmental programming, hormonal signaling (jasmonic acid, salicylic acid), and stress adaptation (biotic challenges, abiotic stresses) [20]. Our *cis*-element annotation categorized conserved motifs into four functional clusters: growth regulation (17 elements, e.g., auxin-responsive elements), light sensing (7 elements, e.g., Box4), stress adaptation (12 elements, e.g., AREs), and hormonal signaling (9 elements, e.g., GARE-motifs) (Figure 5). Notable differences exist regarding both the types and quantities of elements contained within the gene promoters of the *Gossypium TGA* family members. However, it was observed that members belonging to the same evolutionary branch generally share similar cis-acting elements. All members’ promoters—except for *GaTGA5*—contain AREs (Antioxidant Response Elements), which are implicated in plant antioxidation processes. Furthermore, nearly all members of the cotton *TGA* family possess photoresponse-related elements (such as Box4) within their promoters, suggesting that expression of these genes is induced by light exposure. Additionally, most members contain gibberellin response elements (including P-boxes and GARE motifs), indicating their involvement in gibberellin signaling pathways. Moreover, many members’ promoters include JA and SA response elements along with abscisic acid and auxin response elements. This suggests that *TGA* genes broadly function to regulate cotton growth and development while also playing roles in resisting biotic and abiotic stresses as well as participating in signal transduction processes within *Gossypium*.

### 3.6. Expression Analysis of TGA Gene Family in G. hirsutum

**Figure 6:**
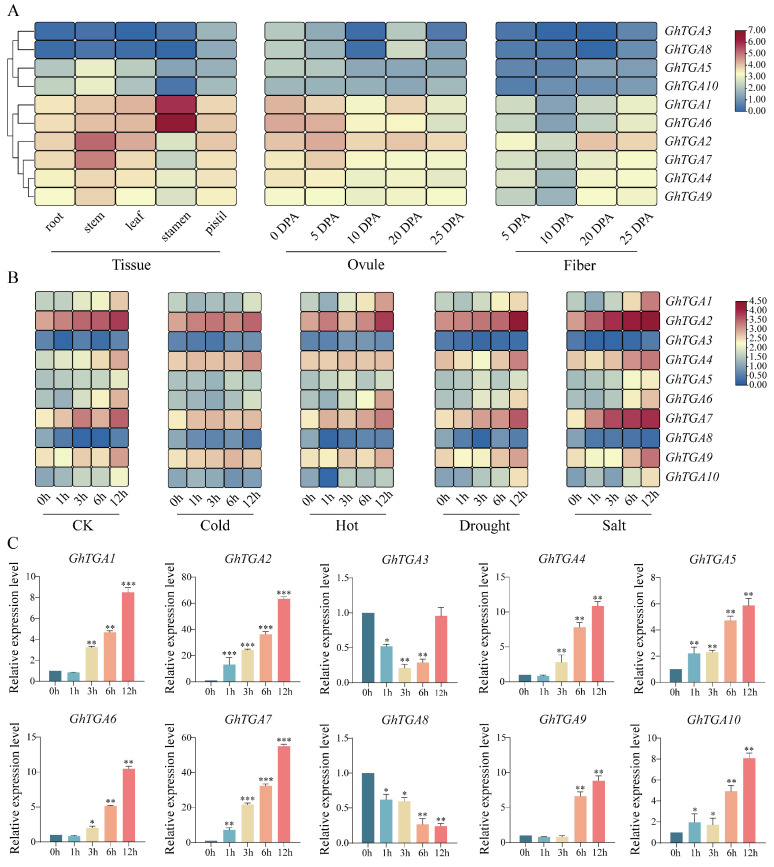
Tissue specific expression and stress-induced expression profile of *TGA* gene family in *G*. *hirsutum*. (**A**) Tissue-specific expression profile of *GhTGAs*. (**B**) Expression profiles of *GhTGAs* under cold, heat, drought, and salt stress. (**C**) Differential expression profiles of *GhTGA* genes under salt stress conditions. The relative expression levels were quantified using the 2^−ΔΔCt^ method with three biological replicates, and normalized to the *GhUBQ6* internal control. Data are presented as mean ± standard deviations from three independent replicates. Statistically significant differences are indicated by asterisks (* *p* < 0.05, ** *p* < 0.01, *** *p* < 0.001) in comparison to the respective NaCl treatment at 0 h.

To comprehensively decipher the spatiotemporal expression dynamics and functional characteristics of TGA transcription factors in *G. hirsutum*, we established a multidimensional expression atlas through systematic integration of publicly available RNA-seq datasets spanning multiple organ-specific contexts. Expression patterns of *GhTGA* homologous genes were analyzed in key tissues, including vegetative (roots, stems, leaves) and reproductive organs (stamens, pistils), ovules at 0, 5, 10, 20, and 25 days post-anthesis, and fiber development from 5 to 25 DPA at 5-day intervals (Figure 6A). Based on the expression profiles observed: *GhTGA3* and *GhTGA8* showed low expression levels (mean FPKM < 1) across all tissues; *GhTGA5* and *GhTGA10* demonstrated higher expression levels specifically in the stem; both *GhTGA1* and *GhTGA6* were predominantly expressed in stamens while also showing high expression in other tissues. Notably, *GhTGA2* was found to be expressed across all tissues—characterizing it as a broad-spectrum gene—particularly prominent during the development stages of cotton fibers at both 20 DPA and 25 DPA. Furthermore, *GhTGA7*, *GhTGA4*, and *GhTGA9* were primarily expressed in stems but also detected in cotton fibers albeit at lower levels.

According to the transcriptome data obtained under various stress conditions, including cold stress, heat stress, drought stress, and salt stress (Figure 6B), it was observed that *GhTGA2*, *GhTGA7*, *GhTGA4*, and *GhTGA9* were generally expressed at high levels across all types of stress. Furthermore, the expression levels of these genes exhibited a gradual increase with prolonged exposure to each type of stress. The expression profile indicates that the *TGA* gene family in *G. hirsutum* plays a significant role in conferring resistance to salt stress, with the *GhTGA2* gene demonstrating the highest expression level specifically under salt-stress conditions. Consequently, we selected *GhTGA2* as the candidate gene for RT-qPCR expression verification at time points of 1 h, 3 h, 6 h, and 12 h during salt stress; notably, our results aligned well with those derived from transcriptome analysis (Figure 6C).

### 3.7. Subcellular Localization of GhTGA2

**Figure 7:**
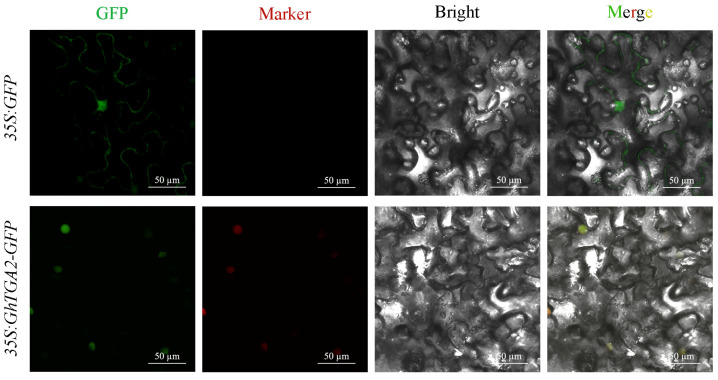
Subcellular localization of *GhTGA2* in *N*. *benthamiana* leaves was analyzed using the empty vector (*35S:GFP*), fusion vector (*35S:GhTGA2-GFP*), and nuclear marker (*pCAMBIA1300-35S-mCherry-NLS*) through infiltration of 4-week-old *N. benthamiana* plants. The images from left to right represent the GFP, Marker, Bright, and Merge channels, respectively. Green fluorescence corresponds to GFP signals, red fluorescence indicates nuclear-localized marker signals, and the merged yellow fluorescence demonstrates the co-localization of *GhTGA2* with the nuclear marker. Scale bar = 50 µm.

To investigate the expression pattern of the *GhTGA2* in cells, we performed transient expression assays using *GhTGA2-GFP* fusion constructs in *Nicotiana benthamiana* epidermal cells. As illustrated in Figure 7, the empty vector exhibits green fluorescence both at the cell membrane and within the nucleus of *N. benthamiana* leaves. In contrast, the *GhTGA2* is primarily localized to the nucleus, aligning with predictions regarding its function and subcellular localization as a transcription factor. This finding suggests that the *GhTGA2* may play a significant role within the nuclear environment.

### 3.8. GhTGA2-Silenced Cotton Plants Showed High Sensitivity to Salt Stress

**Figure 8:**
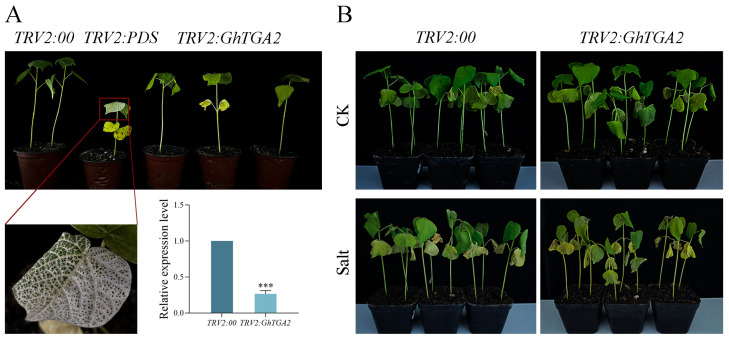
Verification of salt stress tolerance function of *GhTGA2* gene. (**A**) Albino phenotype of the *TRV2:GhPDS* treatment group and identification of VIGS cotton plants that inhibit *GhTGA2* gene expression. The relative expression levels were quantified using the 2^−ΔΔCt^ method. Three biological replicates were used as samples, and normalization was performed using *GhUBQ6* as the internal reference. The data are presented as the mean ± standard deviation of three independent replicates. Compared with the *TRV2:00* control, statistically significant differences are denoted by asterisks (*** *p* < 0.001). (**B**) Comparison of growth phenotypes of *TRV2:00* and *TRV2:GhTGA2* groups before and after salt stress treatment.

To further investigate the biological function of the *GhTGA2* under salt stress in *G. hirsutum*, we constructed a VIGS vector for *GhTGA2* and silenced this gene to induce specific phenotypes in plants, thereby elucidating the role of *GhTGA2*. The *TRV2:GhTGA2* construct was introduced into *Agrobacterium tumefaciens* strain LBA4404 and subsequently used to infect *G. hirsutum*. Both *TRV2:GhTGA2* and *TRV2:GhPDS* vectors were transformed into cotton, and the development of cotton leaves was monitored for two weeks post-infection. The results indicated that cotton plants infected with *TRV2:GhPDS* exhibited albinism in the veins and margins of their leaves by day 7 post-infection; this albinism progressed to encompass the entire leaf by day 14, achieving a silencing efficiency exceeding 85% (Figure 8A). The expression levels of *TRV2:00* and *TRV2:GhTGA2* were assessed using qRT-PCR, revealing a significant suppression of *GhTGA2* gene expression. This finding confirms the successful establishment of a VIGS system targeting cotton. The gene-silenced plants were then cultured for subsequent functional verification experiments.

Subsequently, we subjected cotton seedlings with *GhTGA2* gene silencing and control seedlings to a salt stress treatment using a 200 mM NaCl solution. An equal volume of water was used for irrigation in the control group, and phenotypic observations were made after 15 days of treatment. Results: As illustrated in Figure 8B, following 15 days of growth under normal conditions, both the control plants and those with *GhTGA2* gene silencing exhibited reduced leaf area under salt stress conditions. The leaves displayed significant chlorosis and yellowing, accompanied by brown spots or patches. Additionally, the plants showed signs of wilting and drooping. Notably, the adverse effects of salt stress were more pronounced in the *GhTGA2* gene-silenced plants compared to the negative control group. Silencing the *GhTGA2* gene in *G. hirsutum* increased its sensitivity to salt stress.

## 4. Discussion

As a distinct phylogenetic clade within the basic leucine zipper (bZIP) transcription factor superfamily, TGA proteins orchestrate indispensable regulatory cascades governing plant developmental and stress adaptation pathways. Studies have demonstrated that members of the *TGA* gene family are typically categorized based on their gene sequences, with different groups fulfilling distinct functions in plants while exhibiting relative conservation. Numerous TGA transcription factors across plant species, particularly *AtTGA* clades I–III, mediate abiotic stress tolerance through coordinated modulation of osmolyte biosynthesis, redox homeostasis mechanisms, and phytohormone signaling networks in response to diverse environmental challenges including water deficit, ionic toxicity, and thermal extremes [45,46]. Their multi-level regulatory mechanisms ensure that plants maintain growth balance under varying environmental conditions and support survival and reproduction. *AtPAN* is involved in the early development of *A. thaliana* flowers by controlling stem cell formation and floral meristem termination. In contrast, *AtTGA9* and *AtTGA10* from group IV regulate later stages of flower development [28,46]. Additionally, some studies indicate that *AtTGA7* plays a significant role in controlling flowering time; loss-of-function mutations in *AtTGA7* result in delayed flowering in *A. thaliana* [47]. Although there has been some exploration into the *TGA* gene family across various plant species, research within the cotton genus remains insufficiently developed—particularly concerning salt stress responses. In this study, we utilized *AtTGAs* from *A. thaliana* as query sequences alongside conserved gene sequence analysis and CDD conserved domain analysis to identify a total of 74 members of the *TGA* gene family across ten cotton varieties. Furthermore, eight *TGA* genes were identified within *T. cacao*—a closely related species. The physicochemical properties of these proteins were subsequently analyzed (Table S1). Their instability and hydrophilicity align with the functional role of TGA as a transcription factor. The members of the *TGA* gene family were designated based on their chromosomal locations, leading to the renaming of 10 *A. thaliana* genes to facilitate subsequent research (Figure 1).

An evolutionary tree was constructed using the TGA protein sequences from *A. thaliana*, *T. cacao*, and *Gossypium*, resulting in five distinct branches. The *TGA* gene family of *A. thaliana* was distributed across four branches: Groups I, III, IV, and V. This family can be further categorized into five specific groups: group 1 includes *AtTGA1* and *AtTGA6*; group 2 comprises *AtTGA5*, *AtTGA7*, and *AtTGA8*; group 3 consists of *AtTGA2* and *AtTGA4*; group 4 encompasses *AtTGA9* and *AtTGA10*; while *AtTGA3* is classified under group 5. These findings are consistent with previous studies on the *TGA* family in *A. thaliana* [8]. Evolutionary analysis showed that the five *TGA* gene branches in *Gossypium* contained 25, 20, 16, 10, and 3 members, respectively. Notably, *GhTGA1/6*, *GhTGA2/7*, and *GhTGA4/9* in *G. hirsutum* were closely related to *AtTGA5/7/8* (Group II) from *A. thaliana*, suggesting a role in plant stress responses (Figure 2). Through collinearity analysis it was determined that *G. hirsutum* exhibited collinearity relationships with nine other *Gossypium* species. This finding indicates a conservation of genetic information as well as a co-evolutionary process among different *Gossypium* species—providing an essential foundation for studying gene functions (Figure 3). Members of the *TGA* family residing within the same evolutionary branch in cotton displayed highly conserved gene sequences and protein domains. This suggests that members from different branches may participate in distinct regulatory pathways to fulfill their respective functions (Figure 4). Phylogenetic comparisons of TGA transcription factor orthologs in angiosperms (*A. thaliana* and *N. tabacum*) revealed that the basic leucine zipper (bZIP) domain is highly conserved, with identical tertiary structures maintained across lineages separated by 150 million years. The bZIP domain is critical for determining DNA binding specificity while also serving as a nuclear localization signal [24,46]. Subcellular localization experiments conducted in *N. benthamiana* cells indicated that the *GhTCA2* gene predominantly resides within the nucleus (Figure 7). The functional significance of the bZIP structural module manifests through its mediation of abscisic acid-dependent signal transduction cascades during plant adaptation to hydric deficits and ionic imbalances, with members such as ABA-responsive element binding factors (*ABFs*) serving as canonical regulators of osmotic stress acclimatization [48]. All TGA proteins identified in cotton possess the DOG1 domain. Previous research has demonstrated that, in rice, the DOG1 domain of *OsDOG1L2* can interact with the salt stress-related transcription factor OsDREB2A, thereby enhancing its activation of downstream salt-tolerant genes such as *OsLEA3* and improving the plant’s overall salt tolerance [19].

Systematic exploration of *cis*-regulatory architectures in the *Gossypium* TGA transcription factor family revealed predominant functional associations with phytohormone signaling networks, environmental stress adaptation, photomorphogenic regulation, and developmental programming modules. The transcriptional activation mechanism mediated by the basic leucine zipper (bZIP) structural motif in TGA factors involves sequence-specific binding to the activating sequence-1 (*as-1*) *cis*-regulatory element of pathogenesis-related gene 1a (*PR-1a*), orchestrating SA-dependent defense signaling transduction in plant immune systems [49,50]. Mechanistic studies further demonstrate that TGA transcriptional regulators engage in dynamic protein partnerships with the *NPR1* signaling hub, which coordinates SA-mediated defense signal integration and modulates the transcriptional crosstalk between SA and JA hormonal pathway [51]. Furthermore, TGA transcription factors can also engage with *ROXY19/GRX480* to negatively regulate the expression of defense genes induced by JA and ethylene (ET) [52]. Functional characterization of the *AaTGA6* transcriptional regulator in *A. annua* demonstrates its dual modulation of SA-mediated sesquiterpene biosynthesis, with molecular interaction studies revealing *NPR1* co-activator-mediated enhancement and *TGA3*-mediated suppression of its promoter binding competence at the *AaERF1* regulatory locus [53]. Therefore, it can be speculated that members of the *Gossypium TGA* gene family may modulate stress responses in cotton through their involvement in hormone signaling pathways. Subsequently, we examined the expression profiles of this gene family in upland cotton and discovered that *GhTGAs* exhibited peak expression levels under salt stress (Figure 6). Molecular analysis via reverse transcription-quantitative PCR (RT-qPCR) demonstrated a significant increase in *GhTGA2* mRNA accumulation under salt stress conditions. Furthermore, its expression level was markedly higher compared to other members of the *GhTGAs* gene family, thereby establishing *GhTGA2* as a central regulator of salt adaptation in the *Gossypium*. Parallel functional genomic investigations showed that Agrobacterium-mediated ectopic expression of *GmTGA26* in Glycine max root cultures triggered pronounced activation of abiotic stress-associated transcripts under ionic stress challenges [31]. We selected *GhTGA2*—characterized by broad expression patterns and high inducibility—as a candidate gene and confirmed its resistance function against salt stress using VIGS gene silencing technology (Figure 8). Under salt stress, the plants with silenced *GhTGA2* genes exhibited significant growth retardation, leaf yellowing and curling, as well as the presence of brownish scars when compared to the control group.

This study presents a comprehensive and systematic analysis of the *Gossypium TGA* gene family, exploring its gene structure, protein characteristics, and the features of *cis*-acting elements within the promoter region. Comparative transcriptomic analyses among *G. hirsutum* varieties identified *GhTGA2* as a key regulatory hub within the *TGA* gene family co-expression modules, displaying significant evolutionary conservation and salt-induced transcriptional prominence under conditions of ionic stress. The function of *GhTGA2* was subsequently validated using VIGS technology. However, the molecular mechanisms by which *GhTGA2* modulates salt stress responses in cotton warrant further investigation.

## 5. Conclusions

In this study, a total of 74 homologous *TGA* sequences were identified in the cotton genome through comprehensive genome-wide analysis, which can be categorized into five distinct groups. Among these, 30 pairs of genes from the *TGA* family members of *G. hirsutum* and *G. barbadense* exhibited collinearity during evolution, indicating a closer evolutionary relationship. Expression profiling and functional validation using VIGS technology confirmed that *GhTGA2* plays a crucial role in salt stress tolerance, showing significant transcriptional regulation under salinity conditions. These results yield innovative perspectives regarding the transcriptional regulation of salinity adaptation mechanisms in cotton while simultaneously positioning *GhTGA2* as a prime candidate for marker-assisted selection in genomic-assisted crop enhancement strategies.

## Data Availability

The original contributions presented in this study are included in the article/Supplementary Materials. Further inquiries can be directed to the corresponding author.

## Supplementary Materials

The following supporting information can be downloaded at https://www.mdpi.com/article/10.3390/genes16101143/s1, Supplementary Table S1: Physicochemical Properties of *TGA* Gene Family Members. Supplementary Table S2: The specific primers of *G. hirsutum TGA* genes. Supplementary Table S3: The Connection between the Database and the Online Website in This Study.
